# Comparison of short-term outcomes between robotic and laparoscopic distal gastrectomy performed by the same surgical team during the same period

**DOI:** 10.3389/fonc.2023.1174396

**Published:** 2023-07-06

**Authors:** Jiliang Shen, Xu Feng, Zheyong Li, Yong Wang

**Affiliations:** Department of General Surgery, Sir Run-Run Shaw Hospital, School of Medical College, Zhejiang University, Hangzhou, China

**Keywords:** gastrectomy, gastric cancer, laparoscopic, robotic, short-term

## Abstract

**Objective:**

To evaluate the short-term outcomes of laparoscopic distal gastrectomy and robotic distal gastrectomy performed during the same period.

**Methods:**

This study enrolled 46 cases of laparoscopic distal gastrectomy and 67 cases of robotic distal gastrectomy that were performed by a single surgeon between April 2020 to October 2021. Baseline characteristics and short-term outcomes of these two groups were then compared. Moreover, the robotic distal gastrectomy group was further divided into two subgroups according to the learning curve. Finally, the baseline characteristics and short-term outcomes of both subgroups were compared with the laparoscopic group, respectively.

**Results:**

The baseline characteristics and short-term outcomes of the LDG group and RDG group were comparable. In contrast, the operation time in the laparoscopic group was significantly shorter than that in the early experience robotic group (191.3 ± 37.6 VS 225.1 ± 49, P=0.001). However, the operation time (191.3 ± 37.6 VS 185.3 ± 25.3, P=0.434) was comparable between the laparoscopic group and the late experience robotic group. Likewise, the bleeding volume was comparable between the laparoscopic and early experience robotic groups. However, bleeding volume was significantly lower in the late experience robotic group compared to that in the laparoscopic group (37.5 ± 18.8 VS 49.2 ± 29.0, P=0.049).

**Conclusions:**

With surgeons stepping into the stable stage of the robotic learning curve, RDG showed a comparable operation time and lower volume of blood loss compared with LDG. Collectively, our study supports the application of robotic distal gastrectomy in patients diagnosed with gastric cancer.

## Introduction

Laparoscopy has been widely used in various surgical fields over the past 3 decades (1–4), including laparoscopic gastrectomy, which has been performed in numerous centers (5, 6). Nevertheless, laparoscopy presents a few shortcomings, such as a 2D field of view and limited operating range. Although the advent of 3D laparoscopy has provided a three-dimensional field of vision, it still does not address the issue of a limited range of operations. Meanwhile, the robotic system is a technologically advanced tool in laparoscopy, offering high-definition 3D vision, easier instrument movement, tremor filtration, and superior ergonomics (7). With its popularity, robotic gastrectomy has garnered increasing attention (8, 9). Indeed, robotic gastrectomy exhibits more benefits than laparoscopic gastrectomy, including faster recovery, milder inflammatory responses, and improved lymphadenectomy (10, 11). Based on the findings of previous studies (12–14), any new operation or surgical technology has a peculiar learning curve. For the majority of surgeons, it may take a long time to accumulate sufficient experience in robotic distal gastrectomy and reach the stable phase of the learning curve. Therefore, comparing laparoscopic and robotic surgical data without taking into account the influence of the learning curve does not seem sufficiently rigorous. Our surgical team began performing robotic radical gastric cancer surgery in April 2020. To better compare the data of laparoscopic and robotic distal gastrectomy, patients who underwent laparoscopic and robotic gastric cancer during the same period (from April 2020 to October 2021) were selected for inclusion in this study. Notably, laparoscopic gastrectomy had already been performed for more than a decade before this period. Owing to the limited number of robotic surgical cases, robotic distal gastrectomy cases were further divided into an early experience group and a late experience group according to the learning curve, which was also reported in our previous study (15). Lastly, these two subgroups were individually compared with the laparoscopic group in order to reach more credible conclusions.

## Methods

### Patients

**Figure 1:**
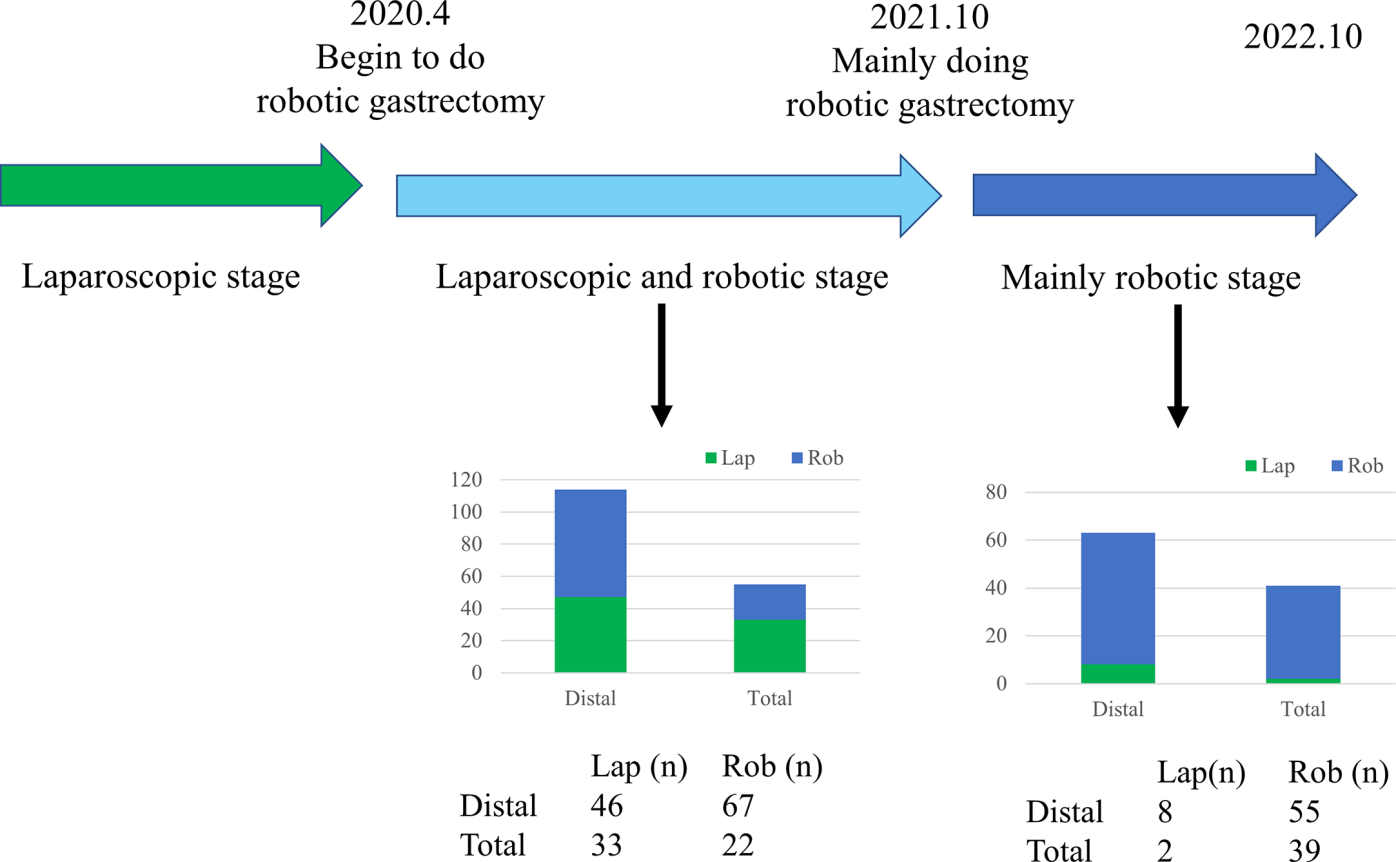
Timeline of laparoscopic, robotic distal, and total gastrectomy performed by the surgical team.

Our surgical team performed the first robotic radical gastric cancer surgery in April 2020. Prior to April 2020, all patients diagnosed with gastric cancer underwent laparoscopic intervention. After October 2020, patients diagnosed with gastric cancer principally underwent treatment with the robotic system (as illustrated in **Figure 1** **)**. Thus, patients diagnosed with gastric cancer (AJCC I-III Stage) from April 2020 to October 2021 were ultimately included in the analysis. Considering that Billroth II and Braun anastomoses were the primary surgical anastomotic methods in our center, patients with other surgical anastomotic methods were excluded. A total of 46 cases of laparoscopic distal gastrectomy and 67 cases of robotic distal gastrectomy were performed by our surgical team. All these patients underwent radical surgical resection.

### Surgeon

All surgical procedures were performed by Professor Wang, who is very familiar with distal gastrectomy with more than 20 years of experience. He has conducted over 1000 cases of laparoscopic distal gastrectomy and initiated robotic distal gastrectomy in April 2020.

### Data collection

Baseline characteristics, including age, gender, BMI, preoperative comorbidities, and preoperative levels of hemoglobin and albumin, were collected. Additionally, data on surgical indicators, including operation time, bleeding volume, postoperative hospital stay, number of lymph node dissections, and major complications, were also gathered.

### Major complications

Major complications, including bleeding, obstruction, and anastomotic leakage, were compared in this study. Bleeding included abdominal bleeding and anastomotic bleeding. Obstruction included intestinal obstruction and anastomotic obstruction. Anastomotic leakage includes duodenal stump fistula and anastomotic fistula. All these complications were indexed as grade 3 or higher (≥3) according to the Extended Clavien-Dindo Classification of Surgical Complications (16).

### Operative technique

STORZ or Olympus laparoscopies were used for laparoscopic distal gastrectomy. The da Vinci Surgical System was used for robotic distal gastrectomy. Either D1 or D2 lymph node dissection was performed in accordance with the Japanese gastric center treatment guidelines. Either Billroth II and Braun reconstruction was carried out in patients of both groups.

### Statistical analysis

Statistical analyses were performed with the SPSS 26.0 (IBM, Armonk, NY) statistical software. Continuous data were presented as mean± standard deviation, whereas categorical data were expressed as frequencies and percentages. The correlation between CUSUM-OT and cases was assessed with linear regression and the coefficient of determination (R2).

## Result

### Baseline characteristics

Professor Wang performed a total of 46 cases of laparoscopic distal gastrectomy and 67 cases of robotic distal gastrectomy from April 2020 to October 2021. The baseline characteristics between the two groups were comparable, as listed in **Table 1**.

**Table 1:** Patient demographics and medical history of robotic and laparoscopic distal gastrectomy groups.

Parameter	RDG(N=67)	LDG(N=46)	P-value
Age, years, mean± SD	65.7±11.2	64.2±11.7	0.467
Sex, female, n (%)	24(35.8%)	21 (45.7%)	0.298
Preoperative HB, g/dL, mean± SD	12.4±2.6	12.1±2.5	0.512
Preoperative ALB, g/L, mean± SD	38.5±6.4	38.6±5.6	0.941
Mean BMI,kg/m2, mean± SD	22.9±4.4	22.1±3.0	0.267
Diabetes, n (%)	5 (7.5%)	3 (6.5%)	0.85
Hypertension, n (%)	17 (25.4%)	9 (19.6%)	0.476
Heart disease, n (%)	3 (4.5%)	2 (4.3%)	0.974
Stage(AJCC 8th Edition)			0.938
I, (%)	28 (41.8%)	21 (45.7%)	
II, (%)	13 (19.4%)	6 (13%)	
III, (%)	26 (38.8%)	19 (41.3%)	

### Surgical outcomes

The short-term outcomes of the two groups were further analyzed, as presented in **Table 2**. There was no significant difference in operative time (191.3± 37.6 vs. 206.1± 44, p=0.065) or surgical blood loss (49.2± 29.0 vs. 48.5± 38.6, p=0.921) between the laparoscopic and robotic groups. Similarly, there was no significant difference in the number of lymph node dissections (34.8± 10.9 vs. 31.4± 8.2, p= 0.057). Furthermore, none of the interventions from either group required conversion to open surgery (0 vs. 0, p=1). Thereafter, major complications, including bleeding, obstruction, and leakage, were analyzed. There were no cases of bleeding in these two groups (0 vs. 0, p=1). Moreover, the rate of obstructions and anastomotic leakage, as well as postoperative hospital stay, were comparable between the two groups.

**Table 2:** Comparison of short-term outcome between robotic and laparoscopic distal gastrectomy groups.

	RDG(N=67)	LDG(N=46)	P-value
Operation time, min, mean± SD	206.1±44	191.3±37.6	0.065
Blood loss, ml, mean± SD	48.5±38.6	49.2±29.0	0.921
Lymph node count, n, mean± SD	31.4±8.2	34.8±10.9	0.057
Open conversion, n (%)	0	0	1
Laparoscopy conversion, n (%)	2 (3.0%)	/	/
Postoperative hospital stay, day, mean± SD	10.9±6.0	11.4± 11.4	0.757
Complication-bleeding, n (%)	0	0	1
Complication-leakage, n (%)	2 (3.0%)	1 (2.2%)	0.794
Complication-obstruction, n (%)	3 (4.5%)	2 (4.3%)	0.974

### Robotic subgroup analysis

Based on our previous research, there is a learning curve in robotic distal gastric surgery. Professor Wang conducted the first robotic surgery in April 2020. According to the learning curve, there is an improvement phase and a stable phase. Differences can be identified in various indicators, such as operative time and blood loss, during each of these phases. Our previous studies have determined that Professor Wang entered the stable phase of the learning curve after performing 35 cases of robotic surgery. At the same time, laparoscopic gastric cancer resection has been performed since 2010 and has been carried out for nearly a decade until April 2020, with a total of over 500 laparoscopic cases conducted. As a result, we postulate that our surgical team already entered the stable phase of the learning curve for laparoscopic distal gastric cancer resection. Therefore, to enable a better comparison of data in the laparoscopic group, the robotic group was divided into the early experience group (1st-35th cases) and the late experience group (36th-67th cases). Baseline characteristics, including age, gender, BMI, AJCC stage, preoperative comorbidities, as well as preoperative hemoglobin and albumin levels, were comparable between the early experience robotic and laparoscopic groups (as portrayed in **Table 3**).

**Table 3:** Patient demographics and medical history of robotic distal gastrectomy (early experience subgroup) and laparoscopic distal gastrectomy group.

Parameter	Early experience(n=35)	LDG(N=46)	P-value
Age, years, mean± SD	63.8±11.5	64.2±11.7	0.871
Sex, female, n (%)	16(45.7%)	21(45.7%)	0.996
Preoperative HB, g/dL, mean± SD	12.5±2.5	12.1±2.5	0.45
Preoperative ALB, g/L, mean± SD	38.3±8.1	38.6±5.6	0.829
Mean BMI,kg/m2, mean± SD	22.8±2.8	22.1±3.0	0.281
Diabetes, (%)	3 (8.6%)	3 (6.5%)	0.731
Hypertension, (%)	8 (22.9%)	9 (19.6%)	0.723
Heart disease, (%)	1 (2.9%)	2 (4.3%)	0.729
Stage (AJCC 8th)			0.838
I, (%)	15 (42.9%)	21 (45.7%)	
II, (%)	8 (22.9%)	6 (13%)	
III, (%)	12 (34.2%)	19 (41.3%)	

Further analysis of the short-term outcomes between the early experience robotic group and the laparoscopic group uncovered that the operation time of the laparoscopic group was significantly shorter than that of the early experience robotic group (191.3± 37.6 vs. 225.1± 49, p= 0.001). Contrastingly, blood loss volume (49.2± 29.0 vs. 58.5± 48.4, p= 0.281) and the number of lymph node dissections (34.8± 10.9 vs. 31.6± 8.2, p= 0.154) were similar. Other indicators, such as the rate of conversion to open surgery, postoperative hospital stay, and major complications, were comparable between the two groups (as shown in **Table 4**).

**Table 4:** Comparison of short-term outcome between robotic distal gastrectomy (early experience subgroup) and laparoscopic distal gastrectomy group.

	Early experience(n=35)	LDG(N=46)	P-value
Operation time, min, mean± SD	225.1±49	191.3±37.6	**0.001**
Blood loss, ml, mean± SD	58.5±48.4	49.2±29.0	0.281
Lymph node count, n, mean± SD	31.6±8.2	34.8±10.9	0.154
Open conversion, n (%)	0	0	1
Laparoscopy conversion, n (%)	1 (2.9%)	/	/
Postoperative hospital stay, day, mean± SD	9.8±3.3	11.4±11.4	0.421
Complication-bleeding, n (%)	0	0	1
Complication-leakage, n (%)	1 (2.9%)	1 (2.2%)	0.847
Complication-obstruction, n (%)	1 (2.9%)	2 (4.3%)	0.729

Bold values means P < 0.05.

Besides, there was no significant difference in baseline characteristics between the late-experience robotic and laparoscopic groups (as displayed in **Table 5**). On the one hand, subgroup analysis of surgical indicators in the late-experience robotic and laparoscopic groups revealed that bleeding volume was higher in the laparoscopic group compared with that in the late-experience robotic group (49.2± 29.0 vs. 37.5± 18.8, p= 0.049). On the other hand, the operation time (191.3± 37.6 vs. 185.3± 25.3, p= 0.434) and the number of lymphatic node dissections (34.8± 10.9 vs. 31.6± 8.2, p= 0.154) were comparable in the laparoscopic group and late experience robotic groups. Additionally, there was no significant difference in the rate of conversion to open surgery, postoperative hospital stay, or major complications between the two groups (as outlined in **Table 6**).

**Table 5:** Patient demographics and medical history of robotic distal gastrectomy (late experience subgroup) and laparoscopic distal gastrectomy group.

Parameter	Late experience(n=32)	LDG(N=46)	P-value
Age, years, mean± SD	68±10.6	64.2±11.7	0.147
Sex, female, n (%)	8 (25.0%)	21(45.7%)	**0.065**
Preoperative HB, g/dL, mean± SD	12.3±2.8	12.1±2.5	0.726
Preoperative ALB, g/L, mean± SD	38.7±3.9	38.6±5.6	0.878
Mean BMI,kg/m2, mean± SD	23.1±5.7	22.1±3.0	0.331
Diabetes, (%)	2 (6.3%)	3 (6.5%)	0.962
Hypertension, (%)	9 (28.1%)	9 (19.6%)	0.384
Heart disease, (%)	2 (6.3%)	2 (4.3%)	0.712
Stage (AJCC 8th)			0.73
I, (%)	13 (40.6%)	21 (45.7%)	
II, (%)	5 (15.6%)	6 (13%)	
III, (%)	14 (43.8%)	19 (41.3%)	

Bold values means P < 0.05.

**Table 6:** Comparison of short-term outcome between robotic distal gastrectomy (late experience subgroup) and laparoscopic distal gastrectomy group.

	Late experience(n=32)	LDG(N=46)	P-value
Operation time, min, mean± SD	185.3±25.3	191.3±37.6	0.434
Blood loss, ml, mean± SD	37.5±18.8	49.2±29.0	**0.049**
Lymph node count, n, mean± SD	31.1±8.3	34.8±10.9	0.103
Open conversion, n (%)	0	0	1
Laparoscopy conversion, n (%)	1 (3.1%)	/	/
Postoperative hospital stay, day, mean± SD	12.1±7.8	11.4±11.4	0.767
Complication-bleeding, n (%)	0	0	1
Complication-leakage, n (%)	1 (3.1%)	1 (2.2%)	0.797
Complication-obstruction, n (%)	2 (6.3%)	2 (4.3%)	0.712

Bold values means P < 0.05.

## Discussion

With advancements in laparoscopic technology over the past 3 decades, an increasing number of surgical procedures have been accomplished through laparoscopic surgery. Indeed, laparoscopic gastrectomy has become a routine procedure (17, 18). Compared to open gastrectomy, laparoscopic gastrectomy is less invasive, has a shorter recovery period, and has a comparable prognosis to open surgery (19). Over the past decade, robotic gastrectomy has seen a progressive increase in development and acceptance. Earlier studies have asserted that robotic gastrectomy outperforms laparoscopy in minimizing the inflammatory response and facilitating lymph node dissection (11, 20). However, robotic gastrectomy also has some drawbacks, such as a higher cost (21), but prior studies have demonstrated that the remaining hospital cost of robotic gastrectomy after deducting the cost of the surgical modality is lower than that of laparoscopic gastrectomy (11). The present study was conducted to compare cases of laparoscopic gastrectomy and robotic gastrectomy, which were carried out by the same surgical team during the same period. Given that the first robotic gastrectomy was performed in April 2020 in our center, all laparoscopic gastrectomy cases were also collected from that month onwards.

Previous retrospective studies have compared laparoscopic and robotic gastrectomy cases from different periods, but the perception of surgery and the expertise of surgeons may have varied over time. Therefore, to improve the accuracy of the results, data from laparoscopic and robotic gastrectomies performed during the same period were selected to reach more credible conclusions.

46 cases of laparoscopic distal gastrectomy and 67 cases of robotic distal gastrectomy were performed by Professor Wang from April 2020 to October 2021 in our center. These patients were all diagnosed with gastric cancer and underwent radical surgical resection. The findings exposed that there were no significant differences in baseline characteristics and underlying diseases between the two groups of patients. Similarly, the results demonstrated no significant differences in operation time, postoperative blood loss, number of lymph node dissections, and rate of postoperative complications.

Laparoscopic gastrectomy has been performed at our center for over a decade. However, robotic gastrectomy was initiated in April 2020 and is associated with a learning curve. Our previous study (15) concluded that Professor Wang’s skills in robotic gastrectomy attained a stable phase after executing 35 cases. Hence, we inferred that surgical outcomes in the two robotic subgroups - namely, the promotion phase and the stable phase of the learning curve - would differ. The robotic distal gastrectomy subgroups were denoted as the ‘early experience robotic group’ (1st-35th cases) and the ‘late experience robotic group’ (36th-67th cases).

Our findings indicated that in the early experience robotic group, the operation time was significantly longer than that of laparoscopic distal gastrectomy. However, the operation time in the late experience robotic group was comparable to that of the laparoscopic group over time. Ergo, we hypothesized that once the stable phase of the learning curve in robotic distal gastrectomy was reached, the operation time would become equivalent to that of laparoscopic distal gastrectomy.

Another crucial parameter for evaluating surgical quality is the volume of blood loss (22, 23). On the one hand, there was no significant difference in terms of blood loss volume between the early experience robotic and laparoscopic groups. On the other hand, blood loss volume was significantly lower in the late experience robotic group compared with that of the laparoscopic group. This observation insinuated that blood loss volume is lower when robotic distal gastrectomy is efficiently performed compared with laparoscopic distal gastrectomy. We postulate that the 3D field of view, superior magnification effect, and wide operating range of the robotic system can better display and manipulate the structure of lymph nodes and vessels, thereby limiting the risk of bleeding during the operation.

The number of lymph node dissections is another significant parameter for evaluating (24, 25) radical resection in gastric cancer. Prior studies have corroborated that it is simpler to remove lymph nodes in robotic gastrectomy than in laparoscopic gastrectomy (26, 27). However, there was no significant difference in the number of lymph node dissections between the laparoscopic group and the robotic group in our study. Besides, the laparoscopic group was also compared with the early-experience robotic and late-experience robotic groups, respectively, and no disparity was noted in the number of lymphatic dissections. Moreover, the AJCC stages of patients in each group were also compared to avoid the impact of stage differences on the number of lymph node dissections, and the results revealed no significant difference in AJCC staging between the groups. In short, the findings of this study implied that robotic technology does not increase the number of lymph node dissections in distal gastrectomy.

Bleeding, obstruction, and anastomotic leakage are common, serious complications in gastrectomy surgery (28, 29) that can be relatively life-threatening to the patient and can significantly prolong the patient’s hospital stay. These three complications were compared, and no significant difference was found between the early experience robotic and laparoscopic groups, nor between the late experience robotic group and the laparoscopic group.

Our study has some limitations that cannot be overlooked. To begin, this was a retrospective study rather than a prospective randomized controlled trial and thus may have been subjected to potential selection bias inherent in retrospective studies. Secondly, the sample size was not sufficiently large to draw definitive conclusions, and further studies are warranted to validate our results. Thirdly, all surgical interventions were performed by the same surgeon in this study, and there can be significant individual differences in the experience and habits of the operating surgeon. Finally, long-term follow-up data could not be collected as the surgical cases were all recently performed.

## Conclusion

In summary, this study compared the baseline characteristics and surgical outcomes of laparoscopic and robotic distal gastrectomy conducted during the same period. Our research findings established that the operative time of robotic distal gastrectomy was similar to that of laparoscopic surgery once the surgeon reached the stable stage of the robotic learning curve. Additionally, robotic distal gastrectomy possesses advantages such as minimal postoperative blood loss. However, further long-term follow-up data on survival and recurrence are necessitated in future studies in order to reach definitive conclusions.

## Data availability statement

The raw data supporting the conclusions of this article will be made available by the authors, without undue reservation.

## Ethics statement

The studies involving human participants were reviewed and approved by the ethic committee of Sir Run-Run Shaw Hospital, Zhejiang University School of Medicine. The patients/participants provided their written informed consent to participate in this study. Written informed consent was obtained from the individual(s) for the publication of any potentially identifiable images or data included in this article.

## Author contributions

JS share first authorship. All authors contributed to the article and approved the submitted version.
